# Health-related quality of life of women with disabilities in relation to their employment status

**DOI:** 10.3325/cmj.2011.52.550

**Published:** 2011-08

**Authors:** Andreja Barišin, Tomislav Benjak, Gorka Vuletić

**Affiliations:** 1 Croatian National Institute of Public Health, Zagreb, Croatia; 2 Department of Psychology, Faculty of Philosophy, University of J.J. Strossmayer, Osijek, Croatia

## Abstract

**Aim:**

To compare the health-related quality of life of unemployed and employed women with disabilities and establish factors affecting their life satisfaction.

**Methods:**

The study included 318 women with disabilities, 160 of whom were employed and 158 unemployed, paired according to age and region of residence. The health-related quality of life was assessed by The World Health Organization Quality of Life questionnaire, and social demographics and factors affecting life satisfaction were collected by a general questionnaire. The factors affecting life satisfaction were defined according to respondents’ statements.

**Results:**

Unemployed women with disabilities had a lower mean score (±standard deviation) on all health-related QoL domains: psychological health (14.52 ± 2.80 vs 15.94 ± 2.55), social relationships (15.12 ± 3.08 vs 16.06 ± 2.69), environment (12.80 ± 2.78 vs 13.87 ± 2.49), as well as on a separate item of self-assessed health (3.33 ± 1.16 vs 3.56 ± 0.92) than their employed counterparts (*P* < 0.01). This disparity was not found only in the domain of physical health. The largest positive impact on life satisfaction in both groups was family.

**Conclusion:**

As disabled women are a particularly vulnerable population group, stressing the importance of employment and family as factors affecting their quality of life may help equalizing opportunities and upgrading the quality of life of all – particularly unemployed women with disabilities.

As estimated by the United Nations (UN), disabilities are far more widespread than believed: one in every 10 inhabitants of the world is to a certain point disabled, accounting for some 450 million persons worldwide (1). Croatia in 2009 registered 511 080 persons with disabilities, which amounts to 11.5% of the overall population, 205 662 (40.2%) of whom were women (2). UN Convention on the Rights of Persons with Disabilities, ratified by Croatia in 2007, defines disabilities as long-term physical, mental, intellectual, or sensory impairments that in interaction with various barriers may hinder persons’ full and effective participation in society on an equal basis with others (3). The right to work and employment is one of the fundamental human rights, which can help equalize opportunities for persons with disabilities and guarantee full and effective social integration (4). According to Croatian Employment Service, the employment rate for disabled persons echoes general hiring trends as the oscillations on the Croatian labor market reflect the global economic crisis.

Recently, considerable research attention has been paid to the impact of employment on the quality of life of women with disabilities, with the term quality of life (QoL), in line with the world health organization’s (WHO) definition of health, referring to psychological, social, and physical well-being of a person and their ability to perform the usual daily activities (5). QoL implies satisfying one’s needs and interests, choice of values, and aspirations in different areas and in different stages of life. Prerequisites for this are full active participation in interaction and communication processes, as well as communication in one’s physical and social environment (3,6,7). Within the scope of the UN Development Program, a study on the quality of life and risk from social exclusion of disabled persons was carried out in Croatia. The principal objective of the Program is to support a balanced development and implementation of social inclusion strategies and policies focusing primarily on the vulnerable groups – first and foremost, persons with disabilities, most threatened by social exclusion and aggravated access to civil, political, and social rights.

A significant connection was proven between the disabled person’s self-assessed position within the society and duration of their unemployment (8). To illustrate, persons who have been unemployed for a longer period believe that they are seen by the society as less valuable and that they feel socially excluded (8). Long-term unemployment is singled out as one of the causes of social exclusion, which is especially hard for women with disabilities (9). Regardless of the severity of their disability, disabled women tend to exhibit less self-confidence and a greater level of social exclusion. The ones who had received more support and love in their families show more self-esteem, which helps them find a job significantly sooner (10). Given that Croatia is one of the poorest countries of Europe, with an increasing unemployment rate (2), there is a great risk of unemployment and social exclusion among disabled women (11). This study is carried out as one of the preventing measures for discrimination of women with disabilities under the National Strategy for Equalization of Opportunities for Persons with Disabilities (4). The aim of this study is to compare the health-related quality of life of unemployed and employed women with disabilities, establish the factors affecting their personal life satisfaction, and give guidelines for advancing their quality of life.

## Participants and methods

### Participants

The study included 160 employed and 158 unemployed women with disabilities. The sample was not randomly selected from an open population, but we included only women who voluntary answered the questionnaire. It was initially planned to match the women according to age, residence, marital, and professional background. However, due to difficulties with data collection, only age and residence could be matched. The planned number of respondents for the control group of employed women with disability was recruited through the Croatian Union of Physically Disabled Persons Associations, Croatian Association of the Blind, Association of Multiple Sclerosis Societies of Croatia, and the web-based employment service Moj Posao. Unemployed women with disability were recruited in cooperation with the Croatian Employment Service and their branch offices, which conducted the survey in all counties. The associations were asked to inform all their members interested in participation, while the Croatian Employment Service was asked to include all registered unemployed women with disability. Implementation coordinators were chosen regionally to distribute the questionnaires to their members and were trained by the researchers on the manner of data collection. The focus was placed on independent, truthful, anonymous, and suggestion-free filling out. In special cases, meetings were organized with interested women with disability, at which researchers further clarified how to fill out questionnaires. In all other cases, questionnaires were sent by mail to coordinators, who after telephone reminders by researchers, sent filled out questionnaires four months later. One thousand members of the associations were informed about the study, 160 (16% response rate) of whom responded. Croatian Employment Service was sent 170 questionnaires, 158 of which were filled out by unemployed women with disability (93% response rate). The study was carried out between April 2008 and April 2009.

### Methods

The questionnaire consisted of three parts: socio-demographic data, two open-answer questions on reasons for satisfaction and reasons for dissatisfaction with life, and The World Health Organization Quality of Life questionnaire (WHOQOL-BREF) (12). WHOQOL-BREF comprises 26 items and is used to multidimensionally assess the quality of life. It measures QoL by encompassing 4 domains: 1) physical health, 2) psychological health, 3) social relationships, and 4) environment, as well as two general questions: health and QoL self-assessment. Physical health domain comprises activities of daily living, dependence on medicinal substances and medical aids, energy and fatigue, mobility, pain and discomfort, sleep and rest, and work capacity. Psychological domain covers bodily image and appearance, negative feelings, positive feelings, self-esteem, spirituality, religion, and personal beliefs, thinking, learning, memory, and concentration. Social relationships domain includes personal relationships, social support, and sexual activity. Environment domain contains financial resources, freedom, physical safety and security, health and social care: accessibility and quality, home environment, opportunities for acquiring new information and skills, participation in and opportunities for recreation and leisure activities, physical environment (pollution, noise, traffic, climate), and transport. Domain results were obtained by combining 24 questionnaire scales. Units of general quality of life and general health were analyzed separately. The questionnaire had good metric characteristics; Cronbach α coefficients for physical, psychological, and environmental domains were above 0.75 and 0.68 for social relationships domain (12).

### Statistical analysis

Descriptive statistics was used to present data on age, family status, and education level of participants (mean and standard deviation for quantitative continuous variable, percentage for categorical variables). Original results from the WHOQOL-BREF questionnaire were transformed according to authors' algorithm, which yielded four health domains. Domain scores were additionally transformed into a scale of 0-100, in a form of percentage of scale maximum for the purpose of better result interpretation and comparison, but the statistical analyses were performed on original scores. *t*-test was used for testing the difference between groups and the difference in QOL according to relationship status. Spearman correlation coefficient was used to test the correlation between WHOQOL-BREF domains and education level (ordinal variable). Statistical analysis was performed using statistical software Statistica, version 7.1 (13).

## Results

The study included 318 women with disabilities, 160 of whom were employed and 158 unemployed. The median (range) age of employed women was 40.5 years (18-73 years) and that of unemployed women was 35.9 years (19-72 years). Two unemployed and unmarried respondents were excluded from the study because they sent poorly filled out questionnaires out of schedule. The study covered different types of physical disability; the inclusion criterion was the ability to fill out the questionnaire independently and properly. Respondents with mental retardation were excluded. The majority of employed women were married (51.9%), as opposed to the unemployed, who were mostly unmarried (58.8%). There were 62.5% of employed women and 77.5% of unemployed women with high school education, and 31.9% of the employed and only 6.9% of unemployed women with university education (Table 1). Since the two groups differed according to education and relationship status, we tested if there was a significant relationship between QoL domains and those variables. There was no difference in QoL between women living with a partner and single women (*P* > 0.05). Education level was not significantly correlated with any of health-related QoL domains in either group of women (Table 2). Unemployed women showed significantly lower health-related QoL on three domains: psychological health, social relationships, and environment, while no significant difference between unemployed and employed women was detected in physical health. The most notable dissimilarity in percentage of the scale maximum (cca. 10.0%) between the studied groups was in the domain of psychological health. Unemployed women rated their overall quality of life significantly lower than employed women. However, two groups of women did not differ in satisfaction with their health in general (Table 3).

**Table 1:** Demographic characteristics of employed (N = 160) and unemployed (N = 158) study participants

Characteristic	Employed women, n (%)	Unemployed women, n (%)
Education level:		
without school	1 (0.6)	3 (1.9)
primary	6 (3.8)	21 (13.1)
secondary	100 (62.5)	124 (77.5)
university	51 (31.9)	11 (6.9)
special education	2 (1.3)	1 (0.6)
Family status:		
married	83 (51.9)	66 (41.2)
unmarried	77 (48.1)	94 (58.8)
Age, median (range)	40.5 (18-73)	35.9 (19-72)

**Table 2:** Spearman correlation coefficient and significance level of correlation between education level and quality of life domains in the World Health Organization Quality of Life questionnaire (WHOQOL-BREF)

	WHOQOL-BREF domain			
Education level	physical health	psychological	social relationships	environment
Employed women	-0.052	-0.029	-0.151	0.070
*P*	0.527	0.716	0.062	0.387
Unemployed women	0.048	0.055	0.155	0.057
*P*	0.564	0.493	0.070	0.489

**Table 3:** Quality of life on four domains and self-assessment of the overall quality of life and satisfaction with health, with the test and significance of difference between unemployed and employed group of women

Domain of The WHO Quality of Life questionnaire*	Employment status	N	Percent of scale maximum	Mean ± standard deviation	*t*-test	Degrees of freedom	*P*	Mean difference (95% confidence interval)
Physical health	unemployed	157	64.6	14.33 ± 3.18	-1.745	315	0.082	- 0.590 (-1.26 to 0.08)
	employed	160	68.3	14.92 ± 2.84				
Psychological	unemployed	158	65.8	14.52 ± 2.80	-4.729	316	0.000	-0.590 (-2.01 to -0 .83)
	employed	160	74.7	15.94 ± 2.55				
Social relationships	unemployed	156	69.6	15.12 ± 3.08	-2.887	314	0.004	- 0.939 (-1.58 to - 0.30)
	employed	160	75.4	16.06 ± 2.69				
Environment	unemployed	158	55.0	12.80 ± 2.78	-3.603	316	0.000	-1.066 (-1.65 to - 0.48)
	employed	160	61.7	13.87 ± 2.49				
**Question**								
How would you rate your QoL?	unemployed	158	n/a	3.40 ± 0.87	-3.735	315	0.001	-0.362 (- 0.55 to - 0.17)
	employed	159	n/a	3.76 ± 0.85				
How satisfied are you with your health?	unemployed	156	n/a	3.33 ± 1.16	-1.946	314	0.053	- 0.229 (- 0.46 to 0 .00)
	employed	160	n/a	3.56 ± 0.92	0.07			

*WHO –World Health Organization.

We explored the main factors that contribute to satisfaction and dissatisfaction with life. The majority of unemployed women listed family (41.0%) as an important factor contributing to their satisfaction with life, followed by positive psychological state (11.3%) and health (10.8%). Employed women, however, ordered the factors differently – family issues (31.4%) were followed by the job-related factor (17.0%) and close relationships (12.1%) (Table 4).

**Table 4:** Number of employed (N = 160) and unemployed (N = 158) study participants who identified different factors as contributing to their satisfaction with life and significance of difference between groups*

Factors Factor	Unemployed women, n (%)	Employed women, n (%)
Family	80 (41.0)	70 (31.4)
Positive psychological state	22 (11.3)	16 (7.2)
Health	21 (10.8)	10 (4.5)
Close relationships with family and friends	18 (9.2)	27 (12.1)
Having a job and job-related factors	13 (6.7)	38 (17.0)
Leisure activities	8 (4.1)	9 (4.0)
Acceptance by people	8 (4.1)	0
Social activities	8 (4.1)	10 (4.5)
Partner	6 (3.1)	1 (0.5)
Helping others	5 (2.6)	4 (1.8)
Education	4 (2.1)	1 (0.5)
Autonomy	2 (1.0)	10 (4.5)
Financial status accounting	2 (1.0)	8 (3.6)
Pets	1 (0.5)	2 (0.9)
Religion	1 (0.5)	1 (0.5)
Achievements	1 (0.5)	1 (0.5)
Acceptance by colleagues and society	0	12 (5.4)
Health care	0	2 (0.9)
Money	0	1 (0.5)

*Each participant could identify more than one factor.

Priority factors of life dissatisfaction and reduced quality of life for unemployed women with disabilities were not having a job (37.2%), the government (12.4%), and financial situation (11.0%), while employed women stated social environment (24.5%), health (17.4%), and the government (10.2%) (Table 5).

**Table 5:** Number of employed (N = 160) and unemployed (N = 158) study participants who identified different factors as contributing to their dissatisfaction with life*

Factors	Unemployed women, n (%)	Employed women, n (%)
Not having a job	51 (37.2)	0
Government	17 (12.4)	10 (10.2)
Financial situation	15 (11.0)	7 (7.1)
Social environment	13 (9.5)	24 (24.5)
Health	13 (9.5)	17 (17.4)
Material/housing situation	11 (8.0)	6 (6.1)
Family	5 (3.6)	5 (5.1)
Achievements	4 (2.9)	2 (2.0)
Autonomy	4 (2.9)	2 (2.0)
Psychological state	4 (2.9)	2 (2.0)
Iniquity	1 (0.7)	5 (5.1)
Education	1 (0.7)	0
Relationship with others	1 (0.7)	0
Having an inadequate job	0	7 (7.1)
Health care system	0	7 (7.1)
Physical environment	0	3 (3.1)
Partner	0	2 (2.0)
Insufficient time for oneself	0	1 (1.0)
*Each participant could identify more than one factor.			

## Discussion

Our study showed that unemployed women with disabilities had a lower self-assessed health-related QoL and a significantly lower self-assessed overall QoL than employed women, which confirms that employment is an important determinant of QoL and health.

Employment cannot be the only factor affecting QoL and health; certain socio-demographic variables, such as education level or relationship status also may have an impact (14). However, our results revealed no difference in QoL between women who lived with a partner and those who lived alone. Also, there was no significant correlation between education level and QoL in either group. This deviation from other studies (14,15) can be explained by the youth of respondents who possibly did not yet fully appreciate education level and relationship status or by the negative influence that disability may have on the perception of the quality of life and health, thus nullifying the positive effects of education and relationship status (15).

For all population groups in an unfavorable position on the labor market, including disabled women, employment or reintegration are essential (16) for health and quality of life (17-21). For example, unemployed persons who suffered disability after a burn injury have a lower QoL and more posttraumatic stress disorder symptoms than persons who returned to work after the accident (16). The benefits of work for persons with disabilities include the ability to support their families, earn a social status and respect from their environment, apply their knowledge and skills, as well as advance professionally (22). Our research points to a noteworthy difference between the observed groups in the psychological health domain, where employed women reported 10.0% higher values than their unemployed counterparts. This reaffirms the thesis that employed women with disabilities have a better self-image, more self-respect and confidence than unemployed women with disabilities. The former were shown to be more socially active, generally more adaptable to change, and competent in everyday life, as well as less prone to illness (3). Women with disabilities who had been unemployed for a longer time reported that their basic problem were functioning disorders (22,23), while this study showed certain other problems (aside from unemployment): inadequate societal and governmental care for the disabled, poor economic situation, and financial and general insecurity, despite all the community efforts (activities under the National Strategy for Equalization of Opportunities for Persons with Disabilities, and in organization of disabled persons associations), which try to improve the QoL and provide equal opportunities for this particularly vulnerable population group.

The possible reasons for this are insufficient media support and less information for persons with disabilities about policy and social activities for equalization of opportunities but also scarce and possibly inadequate political and social measures taken to improve their prospects of employment (3). All undertaken measures are, as our study showed, still insufficient to significantly improve the perception of quality of life. Also, the factors affecting the degradation of the control group’s QoL predominantly belong to the sphere of social environment and inadequate working conditions, which suggests that the society should be continually sensitized about the problems of persons with disabilities and the urgency to advance the process of adapting the workplace to their needs. This study gives insight into factors positively affecting the QoL of women with disabilities. Both groups pointed out family as the most important factor affecting life satisfaction on a larger scale. Since according to the Disabilities Registry (24), women with disabilities live with their families and their families are exposed to great stress (25), this is an encouraging finding (26,3).

Successful employment of persons with disabilities has become an important criterion in evaluating the system efficiency in the areas of social security and QoL in every country (23). According to the European Community Household Panel, between 1995 and 2001 thirteen Member Countries were deemed efficient primarily in terms of self-employment of disabled persons (27). According to the Croatian definition, the self-employment of disabled persons means that one or more persons with disabilities start a craft or cooperative, or freelance, agricultural or forestry activity that is registered in a competent registry (2). Experience showed that in this way persons with disability would more likely get self-employed than persons without disability. Self-employment provides flexibility and offers greater adaptability between disability status and work. Also, self-employed people with disability have higher satisfaction with work, type of work, and working conditions than people without disability who work for somebody else (27). Policy makers should encourage self-employment among the population with disabilities so as to increase the level of satisfaction and the number of disabled persons with a job (27). As this study showed that a job and family raise the quality of life of unemployed women with disabilities, these women should be given employment opportunities (primarily self-employment) as well as support for their families. Such a model is protected by the law since state institutions are obliged to hire a minimum of one disabled person for every 35 employees at a suitable workplace (28). In addition to this legal proposition, it is of utmost importance to undertake all measures under the National Strategy for Equalization of Opportunities for Persons with Disabilities, which aim at strengthening families of persons with disabilities. This legislative framework, together with continuous support for their education and participation on the labor market, and support for their families, should guarantee them a good life. The present results are an attempt to help with the realization of these goals.

There are some factors associated with the methodology and design of our study that limit the generalizability of our results. The sample of unemployed women with disabilities was not randomly selected from an open population, but we included only women who voluntary answered the questionnaire. Therefore, health related QoL of unemployed women with disabilities might be even worse than indicated in this study.

In conclusion, employed women with disabilities showed a higher health-related QoL than their unemployed counterparts. As disabled women are a particularly vulnerable population group, stressing the importance of employment and family as factors affecting the quality of life may help equalizing opportunities for and upgrading the QoL of all – unemployed women with disabilities in particular.

## References

[1] Eurostat. Disability and social participation in Europe; theme 3: population and social conditions, European Commission, 2001 edition. Available from: http://www.re-integrate.eu/?i=reintegrate.en.bibliography.8*.* Accessed: July 19, 2011.

[2] Croatian Employment Service. Available from: *http**://* www.hzz.hr*.* Accessed: July 19, 2011.

[3] National Strategy for Equalization of Opportunities for Persons with Disabilities 2007-2015. Zagreb: Croatian Government, Ministry of Families, Veterans' Affairs and Intergenerational Solidarity; 2007.

[4] Rački J. The theory of vocational rehabilitation of persons with disabilities. Zagreb: University of Zagreb, Faculty of Education and Rehabilitation Sciences; 1997.

[5] Murphy B, Herrman H, Hawthorne G, Pinzone T, Evert H. Australian WHOQoL instruments: User’s manual and interpretation guide. Melbourne (Australia): Australian WHOQoL Field Study Centre; 2000.

[6] Second European Conference of Ministers responsible for integration policies for people with disabilities. Improving the quality of life of people with disabilities: enhancing a coherent policy for and through full participation. Strasbourg Cedex; 2003 May 7; Malaga, Spain.

[7] Virkes D. Multimedia communications intended for the visually impaired. Master's thesis. Zagreb: Faculty of Electrical Engineering and Computing; 2004.

[8] United Nations Development. Study on quality of life in the Republic of Croatia. Zagreb: Target Ltd; 2006.

[9] Amato MP, Ponziani G, Rossi F, Liedl CL, Stefanile C, Rossi L (2001). Quality of life in multiple sclerosis: the impact of depression, fatigue and disability.. Mult Scler.

[10] Nosek MA, Hughes RB, Swedlund N, Taylor HB, Swank P (2003). Self-esteem and women with disabilities.. Soc Sci Med.

[11] Eurostat. Euro area and EU27 employment down by 0.8%. Available from: http://epp.eurostat.ec.europa.eu/cache/ITY_PUBLIC/2-15062009-AP/EN/2-15062009-AP-EN.PDF*.* Accessed: July 18, 2011.

[12] WHOQOL-BREF. Introduction, administration, scoring and generic version of the assessment. Programme on mental health. Geneva (Switzerland): WHO; 1996.

[13] STATISTICA, version.7,1. Tulsa (OK): StatSoft, Inc.; 2005.

[14] Doh CS (1986). Education and the quality of life in Korea and the United States: a cross-cultural perspective.. Public Opin Q.

[15] Vuletić G. Health related quality of life and satisfaction with life in Croatia. Proceedings of the 8th Australian Conference on Quality of Life. 2009 Oct 12. Melbourne (Australia). Available from: http://acqol.deakin.edu.au/Conferences/abstracts_papers/2006/index.htm*.* Accessed: July 18, 2011.

[16] Hogg M, Braithwaite M, Bailey M, Kotsimbos T, Wilson JW (2007). Work disability in adults with cystic fibrosis and its relationship to quality of life.. J Cyst Fibros.

[17] Karazman R, Kloimüller I, Geissler H, Karazman-Morawetz I (1999). Effects on work interest, work ability and health by an ergonomic and health training in elderly public urban bus drivers.. Journal Experimental Ageing..

[18] Ilmarinen J (2009). Work ability-a comprehensive concept for occupational health research and prevention.. Scand J Work Environ Health.

[19] Miller A, Dishon S (2006). Health-related quality of life in multiple sclerosis: The impact of disability, gender and employment status.. Qual Life Res.

[20] Chouinard V (2010). Women with disabilities' experiences of government employment assistance in Canada.. Disabil Rehabil.

[21] New Jersey Council on Developmental Disabilities. Health access for women with disabilities; 2002. Available from: http://www.njcdd.org/WomensHealth/health-news1.pdf Accessed: July 21, 2011.

[22] Employability of unemployed persons with disability [in Croatian]. Available from: http://www.undp.hr/upload/file/204/102043/FILENAME/nezaposleni_web.pdf Accessed: July 20, 2011.

[23] Blum R. Education and employment of persons with disabilities. Zagreb: Croatian Employment Service; 2008. Available from: http://www.hzz.hr/DocSlike/Zbornik2.pdf

[24] Croatian Public Health Institute. Computer file: Croatian Disabilities Registry. Zagreb: CPHI; 2009.

[25] Cummins RA (2001). The subjective well-being of people caring for a family member with a severe disability at home: a review.. J Intellect Dev Disabil.

[26] Benjak T (2009). Historical changes in the attitudes of society towards persons with disabilities.. Journal of Civil Society Development in Croatia..

[27] Pagán R (2009). Self-employment among people with disabilities: evidence for Europe.. Disabil Soc.

[28] Vuletic-Mavrinac G. Relationship between depression and anxiety with a subjective assessment of quality of life. Speciality dissertation. Zagreb: Faculty of Philosophy in Zagreb; 2007.

